# Social Vulnerability and Child Food Insecurity in Developed Countries: A Systematic Review

**DOI:** 10.1016/j.advnut.2025.100365

**Published:** 2025-01-10

**Authors:** Liyuwork Mitiku Dana, César Ramos-García, Deborah A Kerr, Jane M Fry, Jeromey Temple, Christina M Pollard

**Affiliations:** 1 School of Population Health, Faculty of Health Science, Curtin University, Bentley, Western Australia, Australia; 2 Division of Health Sciences, Nutritional Assessment and Nutritional Care Laboratory (LECEN), Tonalá University Center, University of Guadalajara, Guadalajara, Mexico; 3 Curtin Health Innovation Research Institute, Faculty of Health Sciences, Curtin University, Perth, Western Australia, Australia; 4 Centre for Health Services and Clinical Research, The University of Hertfordshire, Hatfield, United Kingdom; 5 Demography and Ageing Unit, Melbourne School of Population and Global Health, University of Melbourne, Melbourne, Australia; 6 Enable Institute, Curtin University, Bentley, Western Australia, Australia

**Keywords:** children, developed economies, food security, systematic review, social vulnerability, socio-ecological model

## Abstract

Food insecurity (FI) is a serious public health concern in economically developed countries, mainly due to unequal resource distribution. Identifying social vulnerability factors [i.e., characteristics of a person or group regarding their capacity to anticipate, cope with, resist, and recover from the effects of child FI (CFI)] and their positive or negative relationship with CFI is important to support targeted action with a scale and intensity that is proportionate to the level of disadvantage. This review aimed to systematically and comprehensively identify key social vulnerability contributors to CFI in economically developed countries and discuss the factors in the context of the socio-ecological model. Five research databases were searched for observational studies published in 2000 assessing social vulnerability factors related to FI in children residing in developed countries. Data screening and extraction were independently conducted by 2 reviewers who recorded factors related to CFI. The QualSyst tool was used to assess risk of bias. From the studies identified (*N* = 5689), 49 articles, predominantly from the United States and Canada, met the inclusion criteria. The identified social vulnerability factors associated with CFI were grouped into 5 based on the socio-ecological model: *1*) individual child, *2*) parental, *3*) household, *4*) community, and *5*) societal factors. The most frequently reported contributors to CFI were income (household factor). Other social vulnerability factors were identified, including the child’s age, parental depression, household crowdedness, social connection, poverty, and residential instability. The lack of consistent measures to define both social vulnerability and CFI in diverse population subgroups impeded meaningful pooling and interpretation of factors interacting with CFI. Recommendations for future studies are to use comparable measures to estimate the extent and severity of CFI and to investigate the relation between social vulnerability, severity, and trajectories of CFI in developed countries.

This trial was registered at PROSPERO as CRD42022291638.

**Statements of significance**

Along with poverty, a vulnerability in the context of childhood food insecurity (CFI) is complex, with multiple associated factors. This review uniquely identified that although low income and poverty are the main social vulnerability factors related to CFI, there is an array of social vulnerability factors that, if addressed, could significantly reduce the likelihood and severity of CFI. These vulnerability factors include housing, household composition, social engagement, ethnicity and racism, and psychosocial and physical health status. This review is the first to comprehensively examine the key social vulnerability factors associated with food insecurity and its severity among children residing in economically developed countries.

## Introduction

Food security (FS) exists “when all people, at all times, have physical, social, and economic access to sufficient, safe, and nutritious food that meets their dietary needs and food preferences for an active and healthy life” [1]. FS is a fundamental human right, including for children [2,3]. Food insecurity (FI), on the contrary, occurs when food intake is disrupted due to financial or other constraints [4]. The prevalence of FI reported in economically developed countries, including Australia, the United States, the United Kingdom (UK), and Canada, ranges between 4% and 20% [[5], [6], [7], [8], [9]]. Children living in vulnerable households have been identified as a population sub-group at higher risk of FI [10,11]. Because of the unequal distribution of available resources (i.e., unbalanced opportunities create a resource surplus for some and a deficit for others), FI is becoming a serious public health concern in economically developed countries, with a considerable proportion of people struggling to eat adequate amounts of nutritious food every day [12,13].

FI in children, reported as the experience of FI in children or households with children, is associated with a range of costly but preventable health and developmental consequences [[14], [15], [16]]. Children living in FI households are more likely to have poorer health outcomes, including social and mental health (e.g., depression) and developmental and academic outcomes [14,15,[17], [18], [19], [20]]. Higher rates of hospitalization and emergency department visits among children experiencing FI have also been reported to contribute to economic and social burdens and high healthcare costs [21,22]. Furthermore, the likelihood of adverse consequences increases with the levels of severity and persistency of FI [14].

Children residing in vulnerable households are at higher risk of experiencing FI and its consequences [23,24]. Vulnerability refers to a collective measure integrating economic, social, environmental, and political exposures [25]. Social vulnerability is context-specific and can influence the capacity to anticipate, cope with, and recover from the effects of other life challenges, such as child FI (CFI), in the current context [23,26]. Evidence shows that financial hardship is a dominant social vulnerability factor for CFI [[27], [28], [29], [30]]. Vulnerability in the context of CFI is complex, with multiple associated factors, many of which are yet to be defined. Households may move in and out of FI depending on the level of support they obtain [31,32]. For example, low-income families with strong social connections may not experience FI, or at a severe level, as those with no social engagement [[33], [34], [35]]. Identifying social vulnerability drivers and their level of impact on CFI is therefore important to support targeted action with a scale and intensity proportionate to the level of vulnerability. Thus, the relative impact of factors influencing social vulnerability and CFI is of interest to policymakers.

There are several reasons why it is crucial to recognize the social vulnerability factors related to CFI in economically developed countries. First, social vulnerability can be modified through changes in social and economic policies [36,37]. Second, FI prevalence has increased in developed countries over the last decade due to increasing economic inequality due to the inadequacy of social protection systems and social welfare safety nets [38,39]. Reversing this trend is a priority for governments in developed countries. Third, food relief programs, the main response for FI in most developed countries, have short-term benefits without addressing the root causes of FI, such as social vulnerability, and hence fail to eradicate FI [39,40]. Fourthly, some families with children who need food assistance do not utilize services for various reasons, e.g., stigma and shame [13,41,42]. Finally, the food provided by food relief services has been questioned in terms of quality, uniformity, and ability to meet recipients’ preferences, as the available types of food may depend on the donors’ discretion [43,44]. Comprehensively identifying the vulnerability factors related to CFI is, therefore, a key step on the path to making informed decisions and targeted actions to reduce FI in children.

Despite the importance of identifying the social vulnerability factors related to CFI, there is a lack of review articles synthesizing the existing evidence in economically developed countries. Most reviews to date have focused on the consequences of FI. Reviews of FI in children have focused on the prevalence of FI in specific regions (e.g., United States or European countries) or population subgroups (e.g., Hispanic children), and there is a research gap about the impact of social vulnerability on the severity of CFI [45,46]. There are no systematic reviews the authors are aware of that consider the key social vulnerability factors associated with the extent and severity of FI in children in countries with developed economies. This research, therefore, aimed to *1*) comprehensively outline the social vulnerability determinants of the extent and, where possible, the severity of FI in children residing in developed countries and *2*) discuss social vulnerability factors associated with CFI in the context of the socio-ecological model (SEM). This SEM speculates complex interactions across multiple levels of influences (individual child, parents/caregivers, households, community, and societal levels) that are proximal and distal factors, both risk and protective factors that can be drawn on to alleviate CFI [47]. This review also makes recommendations for future research and public health interventions.

## Methods

The systematic review methodology was chosen to identify relevant evidence to address the knowledge gaps relating to social vulnerability factors associated with CFI. The breadth of studies identified in preliminary searching confirmed the methodology as appropriate to meet the research questions [48]. The review aimed to answer 2 questions, “What are the social determinants of FI in children?” (social vulnerability) and “Whether these factors are protective or risk factors of FI in children?”

This systematic literature review was registered with PROSPERO (CRD42022291638) and reported according to the PRISMA guidelines [49].

### Search strategy

**FIGURE 1:**
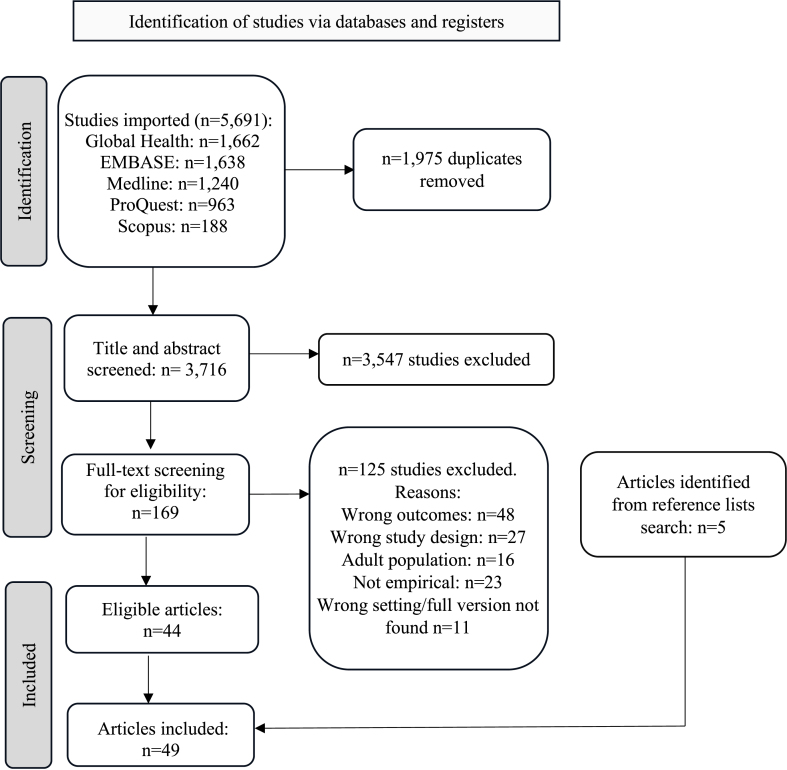
PRISMA flow chart. PRISMA, preferred reporting items for systematic reviews and meta-analyses.

Figure 1 summarizes the process of study identification and inclusion. A search was conducted on 30 June, 2022, across 5 academic databases, including MEDLINE, EMBASE, Scopus, ProQuest, and Global Health (Ovid), and top-up searches were done on 24 June, 2024. The initial search strategy was developed on the basis of key terms included in 4 relevant studies [17,[50], [51], [52]] and encompassed 4 key search concepts: “children,” “food insecurity,” “economically developed countries,” and “social vulnerability” factors (See Supplemental Table 1). These were combined with “AND” and “OR” and grouped to exhaust all the possibilities and to give the search specificity and sensitivity. Additional studies from the reference lists of publications that were eligible for full-text review were included. The PICO (population, intervention, comparator, outcome, study design) structure was used to develop the search strategy.

### Study eligibility and selection

Observational studies (cross-sectional and cohort studies) that investigated the association between FI in children or households with children under 18 y old and social vulnerability factors from 2000 onward in economically developed countries (United Nations country classifications of 2022) and in English language were included. It was acknowledged that FI could be associated with a number of social vulnerability factors and that many of these could mediate or moderate the effect of FI in children. This study is interested in factors directly associated with FI in households with children or the experience of FI reported by children. Studies were eligible if they measured CFI indirectly at the household level (referred to as FI in households with children) or asked children directly about their experiences of FI (referred to as FI in children). CFI nomenclature is used throughout the article, including both approaches; however, the distinction is specified in reporting each study’s details. Studies of children with known health or behavioral conditions and the gray literature (abstracts, conference articles, unpublished material including dissertations, thesis, censuses, and reports from authoritative national and international organizations) were excluded (see Table 1 for detail).

**TABLE 1:** Study eligibility and selection.

Parameter	Inclusion criteria	Exclusion criteria
Population	Children or households with children aged <18 y residing in economically developed countries and without health or behavioral conditions	Adults aged 18+ y, hospitalized/institutionalized setting, children with health or behavioral conditions
Intervention/exposure	Social vulnerability factors of FI in children or households with children	Studies that assessed factors related to FI in children other than social vulnerability factors (e.g., international trade system, politics, war…)
Outcome	FI in children or households with children as an outcome (dependent) variable	FI in children or households with children as the independent variable
Study design	Observational studies (cross-sectional and cohort design) peer-reviewed	Case-control, review articles, qualitative studies, case reports, experimental studies

Abbreviation: FI, food insecurity.

Two authors (LMD and CR-G) independently screened the title, abstract, and keywords (level 1 screening) of the first 100 records and consulted with a second 2 coauthors (CMP and DAK) when assistance was needed to achieve consensus. When decisions could not be made by the title or abstract, the full text was reviewed (level 2 screening). The same process was used to review the full text as outlined by Polanin et al. [53] best practice guidelines.

### Data extraction

Key information was extracted using Covidence software and then exported to an Excel template for further refinement. Items extracted included author(s), publication year, country, sample characteristics [e.g., age of child(ren)], recruitment method, sample size, study design, FI measure, FI prevalence among children, social vulnerability factors assessed (e.g., gender, education, immigration status), and findings about the relationship between social determinants and CFI. Full data extraction was conducted by the lead author (LMD), and 10% of studies were randomly selected, and data were extracted by CR-G to check consistency. Extracted data were reviewed and verified by 2 coauthors (CMP and DAK), and any disagreements were resolved via discussion.

### Evaluation of the quality of the studies

The QualSyst tool was used to evaluate the quality of quantitative studies [54]. Consensus was achieved between 2 authors (LMD and CR-G) who independently assessed the articles based on 11 of the 14 observational studies criteria (excluding randomly assigned and blinding participants), with a maximum possible score of 22 [54]. Eleven checklists against the fulfillment of the criterion for each of the articles were used, with ratings of 2 for yes, 1 for partial, and 0 for not. A conservative minimum cut-off point was taken to select identified studies to be included in this review, with a 75% total score (i.e., a minimum of 16.5 out of a maximum possible score of 22) comparing the overall scores assigned by the 2 reviewers [54].

### Data synthesis and analyses

A meta-analysis could not be conducted as data could not be meaningfully pooled due to heterogeneity of factors (i.e., the diverse determinant of FI in children) and the diversity of sample populations (e.g., households living below the poverty line, asylum seekers, general population). Data were categorized according to the SEM using a narrative assessment [55]. The social vulnerability factors associated with CFI were grouped as *1*) individual child, *2*) parental, *3*) household, *4*) community, and *5*) societal factors. The direction of the relationship between each factor and the extent and severity (marginal, low, or very low) of CFI was represented as either a positive (+ve), negative (−ve), or null (ø) association, and adjusted findings were reported where possible. All studies were included, given they met the minimum cut-off point for the overall scores (16.5 out of 22), regardless of their levels of quality assessment scores that varied between 16.5 and 22.

## Results

### Study characteristics

Table 2 [17,[33], [34], [35],[50], [51], [52],[56], [57], [58], [59], [60], [61], [62], [63], [64], [65], [66], [67], [68], [69], [70], [71], [72], [73], [74], [75], [76], [77], [78], [79], [80], [81], [82], [83], [84], [85], [86], [87], [88], [89], [90], [91], [92], [93], [94], [95], [96], [97]] outlines the characteristics of the 49 studies included in the review. Studies were predominantly conducted in the United States (*n* = 29) and Canada (*n* = 10) and represent a total of 183,829 children, 62,625 families/households with children, and 27,900 child-year observations. There were 38 cross-sectional (including 1 case-control study of precariously housed and homeless people) and 11 longitudinal studies. Quality scores ranged from 17/22 to 22/22 (see Supplemental Table 2).

**TABLE 2:** Characteristics of eligible studies in the systematic review on social vulnerability factors associated with child food insecurity (*n* = 46).

Author, year, country	Sample size	Age of the studied children	Respondents for FI	Study design	Data source	Year of data	FI tool used	FI prevalence
Arteaga et al., 2017, United States [80]	12,700 children	Kindergarten (6.2 y on average)	Caregiver	Cohort	ECLS-K	1998/1999–2010/2011	18-item United States HFSSM	9.7–17.7% (2010–2011) and 12.4–21.7%
Barreiro-Álvarez et al., 2024, Spain [57]	1017 adolescents	11–17 y	Adolescent-parent dyads	Cross-sectional	Survey among adolescents from public and grant-aided schools	2022	9-item Spanish Child Food Security Survey Module (CFSSM-S)	19.2%
Bhargava et al., 2008, United States [81]	7635 children	1, 3, and 5 y	Parents, caregivers	Cohort	ECLS-K	1999–2003	18-item United States HFSSM	7%
Brewer et al., 2019, United States [82]	2700 children Hispanic	4, 8, and 10 y	Parents <400% Federal Poverty Level (FPL)	Cross-sectional	1 child per family from ECLS-K	2011	18-item United States HFSSM	23%
Brewer et al., 2020, United States [83]	1319 HH with children	<16 y	Parent <300% FPL	Cross-sectional	Data from PSID and CDS, national data sets	2014	18-item United States HFSSM	29%
Carter et al., 2012, Canada [69]	1746 mothers with children	4, 8, and 10 y	Mothers	Cohort	Birth registry	1997/1998–2008	3-item Radimer/Cornell hunger and FI	7.1–9.2% at 4 y, 7.6% at 8 y, 7.1% at 10 y
Dave et al., 2024, United States [84]	6403 HHs with children	2–17 y	Mothers	Cross-sectional	NHANES	2013–2016	18-item United States HFSSM	30% HFI (13% CFI)
Denney et al., 2017, United States [75]	3016 females with children	5–10 y	Females are racially and ethnically diverse	Cross-sectional	The California maternal and infant health assessment born during 2003–2007 and data collected (GROWS study)	2012/2013	Researchers developed a 6-item HH FS scale	22.7%
Denney et al., 2020, United States [85]	8600 families with children	Kindergarten (6.2 y on average)	Parents <400% FPL	Cross-sectional	ECLS families’ incomes national data set	2010/2011	The 18-item United States HFSSM	16%
DeRigne et al., 2014, United States [73]	1936 adults with children	0–17 y	1 adult per HH	Cross-sectional	The Making Connections survey in 7 high-poverty community	2008–2011	single item: not enough money to buy food	26.9%
Dhokarh et al., 2011, United States [70]	200 caregivers with children	1–6 y (youngest child)	Low-income female caregivers aged ≥15 y	Cross-sectional	Survey - the acculturation and nutrition needs assessment study	1998–1999	10-item Radimer/Cornell Hunger Scale	40%
Findlay et al., 2013, Canada [33]	1234 parents with children	2–5 y	Parents from HH with children	Cross-sectional	the data were from the Aboriginal children’s survey.	2006	single-item hunger indicator	24.4%
Garg et al., 2015, United States [86]	917 low-income mothers	9–2 y	Mothers <185% FPL	Cohort	Data from the ECLS, birth cohort in the United States (born in 2001 and followed ≤2007)	2001–2007	18-item United States HFSSM	11.8%
Gichunge et al., 2015, Australia [34]	71 refugee HH with a child	<18 y	Primary food preparer in the HH	Cross-sectional	Using a researcher-administered questionnaire	2012	18-item United States HFSSM	18%
Godrich et al., 2017, Australia [50]	219 caregiver-child dyads	9–13 y schoolchildren	Children themselves	Cross-sectional	A caregiver-child dyad survey	2013–2015	CFSSM	20.1%
Huet et al., 2017, Canada [87]	431 HH with children	<18 y	Adults of the last birthday in the HH, regardless of age	Cross-sectional	Survey on randomly selected HH with children	2012–2013	18-item United States HFSSM (1 mo prior)	32.9%
Ip et al., 2015, United States [59]	248 farmworker families with children	Preschool-aged children (2.5–3.5 y)	Latino females with a 3-y-old	Cohort	Survey based on farmworker serving institutions - quarterly food security assessments	2011–2014	Spanish 18-item United States HFSSM	49%
Jolly et al., 2023, UK [78]	75 HHs (138 children)	<18 y	Adults in HH with children	Cross-sectional	Undocumented migrants survey from immigration advice drop-in services	2016	18-item United States HFSSM	94.6% HFI (63.5% VLFS and 75.6% CFI)
Jomaa et al., 2020, United States [88]	365 caregiver-child dyads	Preschool-aged children (4 y on average)	Caregivers	Cross-sectional	SNAP-Ed-Eligible Head Start families	2017/2018	18-item United States HFSSM	37%
Kalil et al., 2008, United States [89]	6068 families with children	Kindergarten (6.3 y on average)	Families <200% FPL	Cross-sectional	ECLS-K	1998–1999	18-item United States HFSSM	6–20%
Kansanga et al., 2022, Canada [35]	21,455 Adults with children	≥12 y	Adults ≥18 y	Cross-sectional	the Canadian community health survey	2017–2018	18-item United States HFSSM	5.15%
Kowalski et al., 2021, United States [68]	496 caregivers with children	Preschool- to adolescent (3–15 y)	Caregivers	Cohort	Recruited from the CHAMP and WCC studies	2017–2020	2-item HH FI Screen	22–25%
Lee et al., 2021, United States [67]	714 parents with children	<18 y	Parents with ≥1 of their own children	Cross-sectional	National Survey Of Homeless Assistance Providers And Clients (NSHAPC) - telephone and mail survey	1996	3-item subset, 8-item child scale	60.7% (12.6% VLFS)
Lippert et al., 2021, United States [90]	714 homeless and precariously housed families (1561 children)	Children under families’ care (≥18 y)	Adult responding person	Cross-sectional	Data are drawn from the NSHAPC	1996	the current population survey (CPS) FI module	61%
Liu et al., 2023, Canada [58]	8416 adolescents	12–17 y	Adolescents	Cross-sectional	Canadian Community Health Survey	2017–2018	18-item United States HFSSM	20.7%
Martin-Fernandez et al., 2018, France [60]	772 homeless families with children sheltered in different facilities	<13 y	Parents aged ≥18 y	Cross-sectional	Face-to-face survey from homeless families sheltered emergency, social rehabilitation, social hostels, and asylum seeker centers.	2013	French 18-item United States HFSSM	53.1%
McIntyre et al., 2000, Canada [71]	13,439 HHs with children (22,831 children)	2–11 y	Families with children	Cross-sectional	Canadian national longitudinal survey of children and youth	1994	Single item (experience of child hungry)	1.2% hunger
McIntyre et al., 2002, Canada [77]	141 lone mothers with ≥2 children <14 y	<14 y	Mothers’ income below Canada’s low-income cut-off	Cross-sectional	4 weekly interviews	1999/2000	Cornell/Radimer	96.5% (23% child hunger over 1 mo)
Melchior et al., 2009, UK [66]	1116 families	5–11 y	Families with young children	Cohort	Data from a register of 1994/1995 twin births in England and Wales	1999/2000–2005/2006	7-item scale USDA	9.7%
Miller et al., 2018, United States [65]	36,302 children lived with biological mothers	Kindergarten and grades 1, 3, 5, and 8	Children, their parents, teachers, and school administrators	Cohort	The ECLS-K cohort	1999/2000–2005/2006	8-child questions from United States HFSSM (CFI)	-
Miller et al., 2014, United States [51]	31,900 from multiple existing data sets	≤17 y (vary depending on the data set): ECLS-B (0–6 y); FFCWS (2–6 y), ECLS-K (5–14 y); and PSID-CDS I and II (3–17 y)	Biological mothers of ≥1 child in the family	Cross-sectional	Multiple existing cohort data sets: ECLS-B; FFCWS, ECLS-K; and PSID-CDS I and II	-	8-item child from United States HFSSM	4.7–8.1% (depending on the data set used)
Morrissey et al., 2016, United States [91]	12,550 children	5.5 y	Parents/caregivers	Cross-sectional	ECLS-K	2010–2011	18-item United States HFSSM	13% HFI (1% CFI)
Nagao-Sato et al., 2021, United States [61]	106 adolescents	10–14 y	Father-mother dyads	Cross-sectional	Baseline data from adolescents and male caregivers who were involved in a community-based intervention program.	2017–2020	2-item screener of 18-item United States HFSSM in Spanish	39% (fathers); 55% (mothers)
Paquin et al., 2021, Canada [74]	2032 HH with children	1.5–13 y	Mothers	Cohort	A population-based birth cohort: 5 mo to 15 y	1998–2013	Single item run out of food	3.6%
Parekh et al., 2021, United States [62]	4312 adults with children	<18 y	Adults living with children	Cross-sectional	Via social media	2020	A 6-item United States HFSSM	14.7%
Potochnick et al., 2019, United States [92]	1466 children (943 Hispanic/Latino HH)	8–16 y	Each child and 1 caregiver	Cohort	Data from 4 major Hispanic/Latino settlement locations	2012–2014	18-item United States HFSSM	33% (10.9% VLFS)
Ramsey et al., 2011, Australia [17]	185 HHs with children	3–17 y	Individuals in HH aged 25–45 y	Cross-sectional	Recruited HHs with children from the most disadvantaged <5% of the census	2009	18-item United States HFSSM	34%
Reesor-Oyer et al., 2021, United States [93]	4897 HH with children	3 and 5 y	Mothers	Cohort	FFCW study born 1998–2000 (wave 3 and 4)	2003–2005	18-item United States HFSSM	15% at T1and 17% at T2)
Rubio et al., 2019, United States [94]	12,035 children	Kindergarten and first grade (7.1 y on average)	Children, parents, and school administrators	Cross-sectional	ECLS-K	2011	18-item United States HFSSM	11.6%
Ruiz-Castell et al., 2015, Canada [63]	292 HH with children	8.5 and 14.5 y	Primary caregiver and child dyads	Cross-sectional	Survey data	2005–2010	4 questions from the 18-item United States HFSSM	27%
Schlichting et al., 2019, New Zealand [76]	6385	0.75 y (9 mo)	Mothers	Cross-sectional	All births in New Zealand from	2007–2010	15-item infant FS index (researchers developed)	43% (16% highly FI)
Sharkey et al., 2011, United States [72]	484 HHs with children	<18 y	Indigenous female health workers in HH food preparation role	Cross-sectional	the colonia HH and community food resource assessment (C-HCFRA)	2009	Radimer/Cornell measures of hunger and FI	61.8% (49% CFI)
Utter et al., 2017, New Zealand [56]	9107 students (2007); 8500 students (2012)	High school	Students	Cross-sectional (2-points)	2 nationally representative surveys of the health and wellbeing of high-school students	2007 and 2012	Single item FS concern item (Researchers developed)	FI concern: 2007: 8%; 2012: 28%
Ward et al., 2019, United States [64]	693 HHs with children	3–5 y	Parents/caregivers and their children	Cross-sectional	The Head Start program parents whose incomes are at ≤100% of poverty	2006	6-Item United States HFSSM	16.1%
Wehler et al., 2004, Canada [95]	220 low-income females (28 y on average) with children at risk of homelessness	Children living with females	Females from low-income homeless and housed	Unmatched case-control for homelessness	The Worcester Family research project	-	A set of 7 dichotomous hunger measure	17%
Wetherill et al., 2021, United States [79]	188 food pantry clients with children	HHs with children accessing food pantries	Only 1 client per HH	Cross-sectional	The food independence, security, and health (FISH) study	2016	18-item United States HFSSM	70.6% (23.3% VLFS)
Willis et al., 2019, United States [52]	1493 adolescent	10–12 grades	Students	Cross-sectional	School survey	2016	Abbreviated 5- items Radimer/Cornell	32.6–58.6% (differed by ethnicity)
Zace et al., 2021, Italy [96]	573 HH with children	1–11 y	Parents of Italians who lived 5 y before pregnancy	Cross-sectional	All the children and their parents visited	2017–2018	18-item United States HFSSM	9.1% CFI
Zhang et al., 2013, United States [97]	27,900 child-year observations	Kindergarten to eighth grade (6.2 y on average in 1999 and 14.3 y in 2009)	Low-income caregivers, schools, and children	Cohort	ECLS-K	1999–2007	18-item United States HFSSM	10.5–14.3% (between 1999 and 2007)

Abbreviations: CFI, child food insecurity; ECLS-B, the Early Childhood Longitudinal Study—Birth cohort; ECLS-K, the Early Childhood Longitudinal Study—Kindergarten cohort; FFCWS, the fragile families and child wellbeing study; FI, food insecurity; HFI, household food insecurity; HH, households; NHANES, National Health and Nutrition Examination Survey; PSID-CDS, the panel study of income dynamics—child development supplement; United States HFSSM, United States household food security survey module; VLFS, very low food security; y, years old; GROWS, geographic research on wellbeing; CHAMP, Creating Healthy Habits Among Maryland Preschoolers; WCC, Wellness Champions for Change; SNAP, Supplemental Nutrition Assistance Program.

Mothers, parents, or guardians reported the experience of CFI in the majority of studies. Eight studies reported the school year rather than the age of the children. Most studies estimated CFI based on household FI level (*n* = 45) and included both girls and boys, and the other 4 studies directly asked children about their experience of FI [52,[56], [57], [58]].

### Measures of FI

CFI instruments varied with 20 studies using the USDA 18-item United States Household FS Survey Module (United States HFSSM) [18,34,35,[57], [58], [59], [60], [61], [62], [63], [64], [65], [66], [67], [68],^98^], including translations in Spanish [59] and French [60]) and 11 studies using a shortened United States HFSSM (the 3-, 6-, or 8-item child scale, 2-item screening questions) [51,57,[61], [62], [63], [64], [65], [66], [67], [68]]. Although the United States HFSSM distinguishes 4 levels of the severity of FI [high FS, marginal FS, low FS (LFS), and very low FS (VLFS)], most studies reported either “food secure” (combining high and marginal FS) or “food insecure” (combining LFS and VLFS) [98,99], with the exception of 6 studies [64,[73], [74], [75], [76],^88^]. Five studies used the 9-item Radimer/Cornell instrument [52,[69], [70], [71], [72]], 4 used a single-item measure [33,71,73,74], and another 4 used a researcher-developed instrument [34,56,75,76].

### Prevalence and severity of CFI

All except 1 study [65] reported the prevalence of CFI, ranging from 1.0% to 96.5% depending on the population group studied (Table 2) [17,[33], [34], [35],[50], [51], [52],[56], [57], [58], [59], [60], [61], [62], [63], [64], [65], [66], [67], [68], [69], [70], [71], [72], [73], [74], [75], [76], [77], [78], [79], [80], [81], [82], [83], [84], [85], [86], [87], [88], [89], [90], [91], [92], [93], [94], [95], [96], [97]]. The highest prevalence (96.5%) was reported by McIntyre et al. [77] (2002), who assessed FI among households headed by lone mothers with ≥2 children under the age of 14 y with incomes below Canada’s low-income cut-off. Jolly et al. [78] (2023) found a prevalence of 94.6% FI among undocumented migrants with children attending a UK immigration advice service, and Wetherill et al. [79] (2021) reported that 71% of United States food pantry clients with children experienced household FI. The lowest prevalence was reported by McIntyre et al. [71] (2000), who found that 1% of families with children aged 2–11 y in the United States experienced FI. Similar rates were reported by Paquin et al. [74] (2021), who studied FI among Canadian mothers with children aged 1.5–13 y (3.6%).

Six studies reported the severity of CFI [64,[73], [74], [75], [76],^88^]. Jolly et al. [78] (2023) reported the highest proportion of severe FI among undocumented migrants with children (63.5% experienced VLFS) [75]. Wetherill et al. [79] (2021) found that 23.3% of food pantry clients with children in the United States experienced VLFS [76].

### Social vulnerability and CFI according to the SEM

**FIGURE 2:**
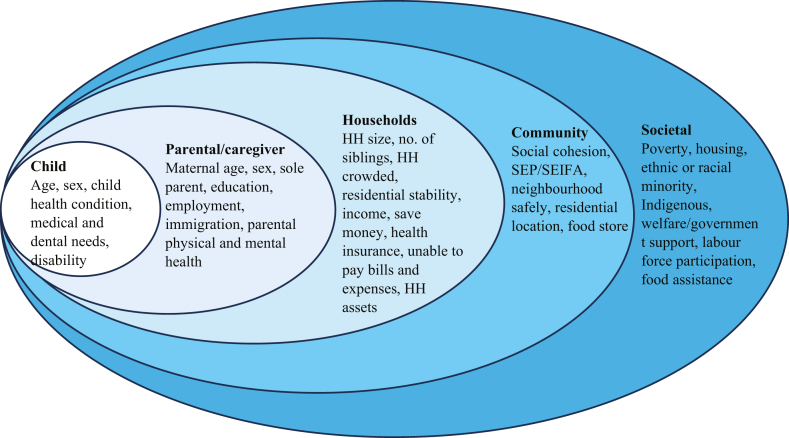
Socio-ecological model of social vulnerability factors and childhood food insecurity. HH, household; SEIFA, socio-economic indexes for areas; SES, socio-economic status. Adapted from reference McLeroy, 1988 [47] with permission.

Table 3 [17,[33], [34], [35],[50], [51], [52],[56], [57], [58], [59], [60], [61], [62], [63], [64], [65], [66], [67], [68], [69], [70], [71], [72], [73], [74], [75], [76], [77], [78], [79], [80], [81], [82], [83], [84], [85], [86], [87],[89], [90], [91], [92], [93], [94], [95], [96],^98^] details the key risk and protective factors associated with CFI according to the SEM [47], and Figure 2 illustrates the identified factors in the SEM. This model shows the problem and potential solutions at proximal and distal levels, including individual child factors, parental and household factors as proximal influences, and community and societal factors as distal influences.

**TABLE 3:** Studies examining social vulnerability factors associated with child food insecurity (*n* = 49).

Factors	Authors (year)	Association direction	Social factors related to child FI
Individual child factors			
Child’s age	Carter et al., 2012 [69]; Wehler et al., 2004 [95]; Lee et al., 2021 [67]; Lippert et al., 2021 [90]	+ve	HHs with higher mean child age were more likely to report FI. HHs with children >5 y were more likely to report FI than <5 y children
	Utter et al., 2017 [56]	+ve	Adolescents 14–15 y more likely to report FS concerns than younger students
	Bhargava et al., 2008 [81]	−ve	HHs with younger children were more likely to report FI
	Miller et al., 2014 [51]	ø	The child’s age is no difference
Sex of the child	Willis et al., 2019 [52]	+ve	Adolescent females were more likely to report FI than their male counterparts
Child with disability/health condition	Denney et al., 2020 [85]; DeRigne et al., 2014 [73]; McIntyre et al., 2000 [71]	+ve	HH having a child with a limited health condition are more likely to report FI
Parental factors			
Mother’s age	Arteaga et al., 2017 [80]; Brewer et al., 2019 [82]; Zace et al., 2021 [96]; Schlichting et al., 2019 [76]	−ve	HHs with younger mothers are more likely to report FI
	Denney et al., 2020 [85]; McIntyre et al., 2002 [77]; Miller et al., 2014 [51]	+ve	HH with older mothers are at higher risk of FI and hunger
	Melchior et al., 2009 [66]	ø	Mother’s age has no significant relationship to FI
Adult responding person’s age	DeRigne et al., 2014 [73]; Bhargava et al., 2008 [81]	+ve	FI increases as the age of responding adults increases
Sex of the head of the households	DeRigne et al., 2014 [73]	+ve	Female respondents are more likely to report FI
Parental/maternal employment	Arteaga et al., 2017 [80]; Dhokarh et al., 2011 [70]; Denney et al., 2020 [85]; Huet et al., 2017 [87]; Parekh et al., 2021 [62]; Ruiz-Castell et al., 2015 [63]; Potochnick et al., 2019 [92]; Sharkey et al., 2011 [72]	−ve	HH with unemployed mothers are more likely to report FI than employed mothers
Maternal education	Arteaga et al., 2017 [80]; Denney et al., 2017 [75]; Gichunge et al., 2015 [34]; Huet et al., 2017 [87]; Kalil et al., 2008 [89]; Miller et al., 2014 [51]	−ve	HHs with less maternal education in high school or college are more likely to report FI compared to their counterparts
	Melchior et al., 2009 [66]	ø	HHs with mothers with reading difficulties have higher FI
Parental education	Brewer et al., 2019 [82]; Miller et al., 2018 [51]; Paquin et al., 2021 [74]; Parekh et al., 2021 [62]; Ruiz-Castell et al., 2015 [63]; Morrissey et al., 2016 [91]	−ve	HH with parent education with college or more are less likely to report FI compared to under high school
	Findlay et al., 2013 [33]	−ve	HHs with 1 parent above high school are more likely to report FI than those with 2 parents
Responding person education	DeRigne et al., 2014 [73]		As the responding person’s education levels are higher, HH FI is less likely to be reported
Highest level of education in the household	Liu et al., 2023 [58]	−ve	As the highest education level in the HH, FI is less likely to be reported
Sole parent	Brewer et al., 2019 [82]; Dhokarh et al., 2011 [70]; Carter et al., 2012 [69]; Kalil et al., 2008 [89]; Kansanga et al., 2022 [35]; Lippert et al., 2021 [90]; Liu et al., 2023 [58]; Martin-Fernandez et al., 2018 [60]; McIntyre et al., 2000 [71]; Miller et al., 2014 [51]; Miller et al., 2018 [65]; Paquin et al., 2021 [74]; Willis et al., 2019 [52]; Brewer et al., 2020 [83]; Denney et al., 2020 [85]	+ve	Single-mother/sole-parent families are more likely to report FI than a family with 2 parents
Parental depression	Denney et al., 2020 [85]; Kansanga et al., 2022 [35]; Paquin et al., 2021 [74]; Rubio et al., 2019 [94]		HHs with depressed primary caregivers are likely to report FI (father or mother depression)
	Martin-Fernandez et al., 2018 [60]	+ve; ø	Parental depression is positively related to VLFS but not LFS
Maternal depression	Garg et al., 2015 [86]; Reesor-Oyer et al., 2021 [93]	+ve	Maternal depression at baseline associated with HH FI at baseline and follow-up (longitudinal and concurrent)
	Melchior et al., 2009 [66]; Kalil et al., 2008 [89]; Miller et al., 2018 [65]; Ward et al., 2019 [64]	+ve	HHs with maternal depression and psychosis spectrum disorder are more likely to report FI
Responding to a person’s mental problem	Kansanga et al., 2022 [35]; Lippert et al., 2021 [90]; Lee et al., 2021 [67]	+ve	Poor mental health problem reported by the responding person is associated with HH FI
	Wetherill et al., 2021 [79]	ø	Poor mental health problems (depression and anxiety) are not associated with FI
Parents with disability/health conditions	McIntyre et al., 2000 [71]; Miller et al., 2018 [65]; Rubio et al., 2019 [94]; Wehler et al., 2004 [95]; Bhargava et al., 2008 [81]	+ve	Mothers with poor health and activity limitations are more likely to report FI
	Wetherill et al., 2021 [79]	ø	There was no association between children of parents with poor health and FI in households with children
Parental poor self-rated health	Brewer et al., 2019 [82]; Kansanga et al., 2022 [35]; Brewer et al., 2020 [83]; Denney et al., 2017 [75]	+ve	HHs with mothers in poor self-rated health are more likely to report FI Poor parental self-rated health, more likely to report FI
Household factors			
Income	Arteaga et al., 2017 [80]; Brewer et al., 2020 [83]; Denney et al., 2020 [85]; Denney et al., 2017 [75]; Findlay et al., 2013 [33]; Kalil et al., 2008 [89]; Kansanga et al., 2022 [35]; Kowalski et al., 2021 [68]; Liu et al., 2023 [58]; Melchior et al., 2009 [66]; Miller et al., 2014 [51]; Paquin et al., 2021 [74]; Parekh et al., 2021 [62]; Potochnick et al., 2019 [92]; Ramsey et al., 2011 [17]; Sharkey et al., 2011 [72]; Zace et al., 2021 [96]	−ve	Low HHs income are more likely to experience FI
	Wetherill et al., 2021 [79]	ø	HH income has no significant association with FI
	Nagao-Sato et al., 2021 [61]	−ve	Low-income HHs report FI in both parents
Unable to pay expenses	Wetherill et al., 2021 [79]	ø	HHs unable to pay expenses (mortgage/rent cool or heat) not associated with FI
Ability to save money	Zace et al., 2021 [96]	−ve	HHs who can save money each month are less likely to be at risk of FI
Health insurance	Wetherill et al., 2021 [79]	ø	Adults lacked health insurance not associated with FI
Child health needs to be met	Lippert et al., 2021 [90]	−ve	Children’s medical/dental needs met are less likely to report FI
HH asset	Brewer et al., 2020 [83]	−ve	Households who have <$1000 liquid assets are less likely to report FI. Liquid asset is an asset that can easily be converted into cash within a short amount of time)
Number of family members	Brewer et al., 2020 [83]; Findlay et al., 2013 [33]; Denney et al., 2020 [85]; Carter et al., 2012 [69]; Kalil et al., 2008 [89]; Kansanga et al., 2022 [35]; Potochnick et al., 2019 [92]; Sharkey et al., 2011 [72]	+ve	Larger in the number of people in HH are more likely to report FI
	Melchior et al., 2009 [66]; Miller et al., 2014 [51]	ø	The number of individuals in HH does not have a significant relation to FI
Number of children/siblings	Brewer et al., 2019 [82]; Kansanga et al., 2022 [35]; Liu et al., 2023 [58]; Miller et al., 2014 [51]; Miller et al., 2018 [98]; Paquin et al., 2021 [74]; Wehler et al., 2004 [95]; Zace et al., 2021 [96]; Lee et al., 2021 [67]; Bhargava et al., 2008 [81]; Jolly et al., 2023 [78]	+ve	HH with a larger number of children/larger number of siblings are more likely to report FI
Crowded HH	Ruiz-Castell et al., 2015 [63]	+ve	Crowded HH is more likely to report FI
	Martin-Fernandez et al., 2018 [60]	+ve	HH with ≥3 children are more likely to report very low food security (VLFS- severity form of FI)
Community factors			
Location/safe neighborhood	Denney et al., 2020 [85]; Kansanga et al., 2022 [35]; Willis et al., 2019 [52]; Nagao-Sato et al., 2021 [61]	−ve	HHs in safe neighborhoods for children to play outside are less likely to report FI
	Dave et al., 2024 [84]; Kowalski et al., 2021 [68]; Morrissey et al., 2016 [91]	+ve	Living in urban areas are more likely to report FI
	Parekh et al., 2021 [62]; Zace et al., 2021 [96]	ø	Living in urban areas, compared with rural areas, was not found to be associated with FI. Living at the center of the center is less likely to report FI
Distance to the food store	Sharkey et al., 2011 [72]; Wehler et al., 2004 [95]; Zace et al., 2021 [96]	+ve	Greater distance to their food store and less perceived quality of the community food environment increases FI
SES/ SEIFA/deprivation index	Carter et al., 2012 [69]; Barreiro-Álvarez et al., 2024 [57]; Kalil et al., 2008 [89]; Melchior et al., 2009 [66]; Schlichting e al., 2019 [76]	−ve	Higher SES HHs are less likely to report FI than low or medium SES
	Godrich et al., 2017 [50]		Children living in a location classified as medium SEIFA had the highest FI prevalence than high or low SEIFA
Social cohesion, family connection, or sense of belongingness in the community	Findlay et al., 2013 [33]; Dhokarh et al., 2011 [70]; Denney et al., 2017 [75]; Kansanga et al., 2022 [35]; Gichunge et al., 2015 [34]	−ve	Strong social/community connections or involvement in cultural activities are less likely to report FI
	Carter et al., 2012 [69]; Gichunge et al., 2015 [34]	−ve	HHs with social support are less likely to report FI
	Martin-Fernandez et al., 2018 [60]	−ve	HHs with no contact with family members or relatives are more likely to report VLFS
	Potochnick et al., 2019 [92]	−ve	HHs with strong family functioning are less likely to report FI
	Martin-Fernandez et al., 2018 (60); Willis et al., 2019 [52]	−ve	The absence of family or relative contact is associated with a higher risk of experiencing LFS and VLFS
	Wetherill et al., 2021 [79]; Jolly et al., 2023 [78]	ø	Perceived social support is not associated with FI. In a study from the UK done among undocumented immigrants, those receiving support from friends and family had a lower risk of FI than government support but not statistically significant
	Nagao-Sato et al., 2021 [61]	ø	Family stress does not have a significant difference in HH FI, as reported by both father and mother. Family stress was assessed using 3 questions about the importance of family relations, conflict between personal and family goals, and individualism among family members
Societal factors			
Poverty/economic stress	Brewer et al., 2019 [82]; Kowalski et al., 2021 [68]; Miller et al., 2018 [65]; Morrissey et al., 2016 [91]		A higher HH poverty level is positively associated with FI
	Potochnick et al., 2019 [92]; Zace et al., 2021 [96]	+ve	Economic stress/deterioration is positively associated with FI
Job loss	Kowalski et al., 2021 [68]; McIntyre et al., 2000 [77]	+ve	Sudden job loss, looking for a job, and reduced hours were associated with an increased risk of FI
Temporary/seasonal worker	Lippert et al., 2021 [90]; Lee et al., 2021 [67]; Ip et al., 2015 [59]	−ve	Temporary or seasonal workers are more likely to report FI than steady or nonworkers
Mother labor force	Arteaga, 2017 [80]	−ve	HHs with mothers not in the labor force are more likely to report FI than in the labor force
Welfare/government support recipient	Arteaga et al., 2017 [80]; Godrich et al., 2017 [50]; McIntyre et al., 2000 [71]; Miller et al., 2018 [65]; Ruiz-Castell et al., 2015 [63]	+ve	Welfare-dependent HH are more likely to experience FI
	Kowalski et al., 2021 [68]	−ve	Support payment during COVID-19 reduced risk of FI
Stamp duty users/food assistants use	Dhokarh et al., 2011 [70]; Kalil et al., 2008 [89]	+ve	Households with monthly food stamps lasting less than the whole month are more likely to report FI compared to those who do not use food stamps
	DeRigne et al., 2014 [73]; Liu et al., 2023 [58]; Miller et al., 2018 [65]	+ve	Food stamps HH receipts in the past 12 mo were more likely to report FI compared to those who did not use
	Lippert et al., 2021 [90]; Lee et al., 2021 [67]	−ve	Among homeless and precariously housed children, stamp users are less likely to report FI
	Nagao-Sato et al., 2021 [61]; Wetherill et al., 2021 [79]	ø	Current participation in food assistance programs was no significant difference in FI reported by both father and mother
	Sharkey et al., 2011 [72]; Wetherill et al., 2021 [79]	−ve	Receiving food assistance reduced the severity of FI
School/daycare meal participation	Kowalski et al., 2021 [68]	−ve	Continued school-meal participation was associated with a decreased risk of FI
	Lee et al., 2021 [67]	−ve	Children in daycare/preschool/school are less likely to report FI
Homeownership	Findlay et al., 2013 [33]; Liu et al., 2023 [58]	−ve	Homes owned less likely to report FI
Residential stability	Denney et al., 2020 [85]; Denney et al., 2017 [75]; Martin-Fernandez et al., 2018 [60]; Wehler et al., 2004 [95]	−ve	More residential stability is less likely to report FI
	Wetherill et al., 2021 [79]	ø	HHs in unstable housing are not associated with FI
Poor housing	Martin-Fernandez et al., 2018 [60]	+ve	Those with poor housing conditions before homelessness are more likely to report FI than those who lived in standard housing
Housing subsidy	Wehler et al., 2004 [95]	+ve	A family’s receipt of a housing subsidy was at higher risk of child hunger but not adult hunger
Parent’s country of birth	Arteaga et al., 2017 [80]; Barreiro-Álvarez et al., 2024 [57]; Miller et al., 2018 [65]; Rubio et al., 2019 [94]; Sharkey et al., 2011 [72]; Denney et al., 2020 [85]; Dhokarh et al., 2011 [70]	+ve	HHs with mothers/parents born outside of the studied developed countries were more likely to experience FI than those parents born in the studied countries (among low-income HHs) - Immigrant children were more likely to report FI than nonimmigrant children
	Ramsey et al., 2011 [17]	−ve	Children with a parent born outside of Australia were less likely to experience FI
Current immigration status	Ip et al., 2015 [59]	+ve	Those who do not have proper immigration documentation were more likely to report FI than those with proper document
Language spoken	Dhokarh et al., 2011 [71]	+ve	Those who speak Spanish only in the United States experienced a higher risk of FI than English speakers
Parent nativity	Findlay et al., 2013 [33]; Garg et al., 2015 [86]; Huet et al., 2017 [87]; Kansanga et al., 2022 [35]; Lippert et al., 2021 [90]; Liu et al., 2023 [58]; McIntyre et al., 2000 [71]; Miller et al., 2014 [51]; Miller et al., 2018 [65]; Willis et al., 2019 [52]; Lee et al., 2021 [67]; Morrissey et al., 2016 [91]; Schlichting et al., 2019 [76]	+ve	HHs with Indigenous backgrounds in the studied countries are more likely to report FI
Racial minority	Kalil et al., 2008 [89]; Kowalski et al., 2021 [68]; Liu et al., 2023 [58]	+ve	African Americans/Black people are more likely to report FI
	Melchior et al., 2009 [66]	ø	No difference
	Miller et al., 2014 [51]	+ve; ø	Mixed result, depending on the data used (4 data sets separately analyzed). Hispanic mothers reported a higher risk of FI ECL-B, ECLS-K, and PSID-CDS, whereas no significant difference reported in FFCWS

The direction of the relationship between each associated factor and child FI (dependent variable) was represented as positive (+ve), negative (−ve), and null (ø) associations.

Abbreviations: ECL-B, the Early Childhood Longitudinal Study—birth cohort; ECLS-K, the Early Childhood Longitudinal Study—kindergarten cohort; FFCWS, the fragile families and child wellbeing study; FI, food insecurity; FS, food security; HH, household; LFS, low food security; PSID-CDS, the panel study of income dynamics—child development supplement; SEIFA, socio-economic indexes for areas; SES, socio-economic status; UK, United Kingdom; VLFS, very low food security.

#### Individual child factors

Eleven studies investigated the association between child-level socio-economic vulnerability factors and CFI [51,56,67,69,71,81,90,95]. Seven studies explored the association between CFI and children’s age with mixed results [51,56,67,69,81,90,95]. Five of 7 reported an increased likelihood of FI among households with older children than younger [56,67,69,90,95], whereas 1 study reported that the probability of experiencing FI decreased as children’s age increased [81]. Miller et al. [51] (2014) found no relationship between a child’s age and household FI. Willis et al. [52] (2019) examined the association between a child’s sex and FI among adolescent students and found that female students were more likely to experience FI than their male counterparts.

Three studies assessed the association between physical health and disability in children and FI, finding that in households with ≥1 child with a disability or chronic health condition, there was a positive association with FI [71,73,85].

#### Parental factors

Over two-thirds of the studies (*n* = 37%) examined parental socio-economic vulnerability factors’ influence on CFI [[33], [34], [35],^51^,^52^,^58^,^62^,^63^,^65^,^66^,[69], [70], [71], [72], [73], [74], [75], [76], [77],[80], [81], [82], [83],^85^,^87^,^89^,^90^,^92^,^96^]). Twelve studies examined the relationship between parental demographic variables and CFI [49,50,63,70,73,74,[77], [78], [79],^81^,^86^,^92^] 8 examined maternal age with mixed results [51,66,76,77,80,82,85,96]. Four of the 8 studies found the likelihood of experiencing FI decreased with increasing maternal age [76,80,82,96], 3 reported the likelihood of FI increased [51,77,85]), and 1 found no significant relationship [66]. Two studies reporting the age of the respondent found a positive relationship between age and experience of FI in households with children [73,81]. Two studies reported an association between FI and the gender of the head of the family; where females were head of the household, they were more likely to report FI than when males were [52,73].

Twenty-five studies investigated the association between parental socio-economic vulnerability and FI in households with children [[33], [34], [35],^51^,^52^,^58^,^62^,^63^,^65^,^66^,[69], [70], [71], [72], [73], [74], [75],^80^,^82^,^83^,^85^,^87^,^89^,^90^,^92^]. Of these, 14 studies investigated the relationship between parental educational attainment and CFI. Seven of the 14 studies found that families where the mother did not have a university degree were more likely to report FI [33,34,51,75,80,87,89]. Melchior et al. [66] (2009) examined mothers’ reading difficulty using the wide-range achievement test [100] and found no association with FI. Five studies reported that parents with college or higher levels of educational attainment were less likely to report FI than those whose highest attainment was high school or lower [62,63,65,74,82]. Liu et al. [58] found that children living in households where the highest level of educational attainment was a postsecondary certificate were less likely to report FI than those in households where secondary school completion was the highest level attained [58]. DeRigne et al. [73] (2014) reported an inverse association between FI and the respondents’ level of education.

The 8 studies that investigated the association between parental employment status and CFI all found a reverse association between maternal or parental employment and household FI [62,63,70,72,80,85,87,92].

Marital status and FI in households with children was assessed in 14 studies, with 13 finding that sole parents (i.e., never married, separated, or widowed) were more likely to report FI than couple families [35,51,52,65,[69], [70], [71],^74^,^82^,^83^,^85^,^89^,^90^]. Martin-Fernandez et al. [60] (2018) reported that the likelihood of severe FI was greater among homeless sole parents.

Fourteen studies assessed psychosocial factors (i.e., parental mental health status) and FI in households with children [35,60,[64], [65], [66], [67],^74^,^79^,^85^,^86^,^89^,^90^,^93^,^94^]. Six investigated the association with maternal depression, with 3 longitudinal studies reporting an association with maternal depression at baseline determined household FI at follow-up [65,86,93], and 3 cross-sectional studies reported a positive association between maternal depression and risk of FI [64,66,89]. The 3 studies investigating the relationship between parental depression and the extent of CFI found a positive association between primary caregivers experiencing depression and CFI [74,85,94]. Martin-Fernandez et al. [60] (2018) assessed parental depression and the level of severity of FI and found that children with parents experiencing depression were more likely to experience VLFS but not LFS. Three studies found that the higher the number of mental health problems, the higher the risk of FI [35,67,90], and Wetherill et al. [79] (2021) did not find this association among United States pantry users.

Ten studies assessed the association between parental disability/poor health and self-rated health and CFI [35,65,71,75,79,[81], [82], [83],^94^,^95^]. Five studies found that children of parents with poor health and activity limitations were more likely to report FI [65,71,81,94,95], whereas Wetherill et al. [79] (2021) found no association. The 4 studies that assessed the poor self-rated health of the respondents found a positive association with FI [35,75,82,83].

#### Household factors

Thirty studies (61%) explored the household levels of socio-economic vulnerability factors on CFI [17,33,35,51,58,[60], [61], [62], [63],[66], [67], [68],^69^,^72^,^74^,^75^,^79^,^80^,^82^,^83^,^85^,^89^,^90^,^92^,^95^,^96^]. Of these, 19 studies assessed income vulnerability, and all but 1 study [79] reported low-income households were more likely to experience FI [17,33,35,51,58,61,62,66,68,72,74,75,80,83,85,89,92,96]. Zace et al. [96] (2021) investigated the “ability to save money,” finding that households with children who saved money were less likely to report FI. Wetherill et al. [79] (2021) found no association between household FI and being unable to pay the mortgage, rent, cool or heat the home [79]. Brewer et al. [83] (2020) found that households with fewer liquid assets (assets that can easily be converted into cash in a short timeframe) were more likely to experience FI. Lippert et al. [90] (2021) found that households meeting children’s medical or dental needs were less likely to experience FI. In contrast, Wetherill et al. [79] (2021), in a United States study, assessed the association between private health insurance and FI in households with children and found no association.

The influence of family composition and household structure on CFI was explored in 18 studies with mixed results [33,35,51,58,60,63,66,67,69,72,74,82,83,85,89,92,95,96]. Eleven studies assessed the total number of family members [33,35,51,63,66,69,72,83,85,89,92] and found the likelihood of experiencing FI increased as the number of people in the household increased according to 8 studies [33,35,69,72,83,85,89,92] and no correlation was found after controlling for covariates in 2 studies [51,66]. Ruiz-Castell et al. [63] (2015) found overcrowded households with children more likely to report FI. Nine studies assessed the number of children or siblings and found that the more children in the household, the higher the likelihood of FI [35,51,58,60,67,74,82,95,96], with Martin-Fernandez et al. [60] (2018) finding that families with 3 or more children had a higher likelihood of experiencing FI than those with 2 or less.

#### Community factors

Ten studies (20%) investigated the association between the community-level socio-economic vulnerability factors and CFI [35,50,52,61,62,68,72,85,95,96]. The distance of the households from the city center, nearest food store, or less perceived access to quality community food environments were positively associated with FI in households with children [72,95,96]. Urban compared with rural residential locations was not associated with CFI after controlling for covariates in 2 studies [62,68]. Households in unsafe neighborhoods were more likely to report CFI [35,52,61,85], as were households in the most disadvantaged socio-economic areas [35,52,85]). Conversely, Godrich et al. [50] (2017) reported that Australian children residing in areas of medium disadvantage had a higher prevalence of FI than those residing in lower or higher areas of disadvantage.

#### Societal factors

Two-thirds of the studies (*n* = 33) assessed the association between societal level factors and CFI [17,33,[50], [51], [52],^57^,[59], [60], [61], [62], [63],[65], [66], [67], [68],[70], [71], [72], [73],^75^,^79^,^80^,^82^,^85^,^87^,[89], [90], [91], [92],[94], [95], [96]]. Six studies assessed poverty levels as a measure of socio-economic vulnerability and found a positive association with FI in households with children, despite the differences in measures of poverty levels [65,68,82,91,92,96]. Four studies used family [65,68,82] or neighborhood poverty [91], and 2 measured economic stress [92,96] as a social vulnerability factor.

Job security and involvement in the labor force associations with CFI were investigated by 5 studies [62,63,70,72,80,85,87,92]. Two studies found a positive association between job loss or reduced working hours and FI in households with children [68,71]. Three studies reported that temporary workers were more likely to experience FI than either steady workers or nonworkers [59,67,90]. The increased likelihood of experiencing FI in households with children accessing welfare or government assistance was reported in 5 studies [50,63,65,71,80]). Kowalski et al. [68] (2021) examined government support during the early stages of the COVID-19 pandemic (e.g., May-August 2020), finding that eligible households who received welfare assistance were less likely to experience FI [68].

Eleven studies investigated the association between food assistance and CFI with mixed results [61,65,67,68,70,72,73,79,89,90]. Three studies found food stamp recipients were more likely to report FI [65,73,89], and Dhokarh et al. [70] (2011) found that those accessing monthly food stamps that did not last the entire month were more likely to experience FI than those not accessing food stamps. Two studies investigated FI among precariously housed and homeless families with children and found food assistance users less likely to report FI [67,90]. Continuous use of before-school and school-meal services reduced risk of FI in 2 studies [67,68]. Sharkey et al. [72] (2021) and Wetherill et al. [79] (2021) examined the severity of FI in children and food assistance use and found that receiving food assistance reduced the severity but not the extent of FI. One study found no association between food assistance use and the extent of FI [61].

Six studies assessed the relationship between housing and CFI [33,60,75,79,85,95]. Four reported that children from households with residential instability (e.g., moved house in the past 12 mo) were more likely to experience FI [60,75,85,95]. Conversely, Wetherill et al. [79] (2021) reported no association between housing instability and FI; however, they defined unstable housing as “temporary” or “no” housing. A larger proportion of children from families receiving housing subsidies experienced FI than those with no subsidies [95]. The association between homeownership and FI in households with children was assessed by Findlay et al. [33] (2013), who found that children of families who owned their homes were less likely to experience FI than those who did not.

Seventeen studies assessed the relationship between racial or ethnic minority group background and FI in households with children with mixed results [17,33,51,52,57,59,[65], [66], [67],[70], [71], [72],^80^,^85^,^87^,^90^,^94^]. Nine studies defined “ethnic minorities” as parents born outside of the studied country [17,57,65,66,70,72,80,85,94]. Seven of 9 studies reported that children from households where parents were born outside of the studied country were more likely to experience FI than their counterparts [57,65,70,72,80,85,94]. Ramsey et al. [17] (2011) found that children with a parent born outside Australia were less likely to experience FI, whereas Melchior et al. [66] (2009) found no relationship between parental ethnicity and FI in UK children.

Indigenous families were classified as a racial minority group in 10 studies, with mixed results [33,51,52,59,67,70,71,85,87,90]. Eight studies reported that Indigenous children were more likely to experience FI than their nonindigenous counterparts [33,51,52,67,71,85,87,90]. Miller et al. [51] (2014) analyzed 4 United States national data sets and found that Indigenous children were more likely to experience FI in 3 and no association in the fourth. Garg et al. [86] (2015) longitudinal United States study found Hispanic mothers had lower odds of experiencing household FI than White non-Hispanic mothers and no difference between mothers born outside and within the United States.

Ip et al. [59] (2015) defined “immigrant families” as having no proper immigration documentation and found that children from these families were more likely to experience FI than children of documented immigrants. Dhokarh et al. [70] (2011) defined families who spoke languages other than English at home as an ethnic minority group and found that those speaking only Spanish in the United States were at a higher risk of experiencing FI.

## Discussion

This systematic review examined the relationship of social vulnerability factors with the extent and level of severity of CFI in the context of the SEM as a guide. As expected, poverty and income were the most widely reported influences of CFI, with children from low-income families reporting a higher prevalence and severity of FI. Importantly, this review identified several protective and amplifying social vulnerability factors associated with CFI, including housing, household composition, and psychosocial and physical health status.

Poverty and income are separate, but related factors are measured differently in the studies reviewed. Income is a household factor that relates to the amount of money earned, whereas poverty is a broader societal factor that incorporates contextual factors such as household size and the cost of living in the area. A cut-off is assigned that denotes “below the poverty line” based on a national standard. There are variations in the measurement of poverty across studies; for example, although the United States studies incorporated the federal poverty level, the studies chose different cut-offs, <400% [82] or <300% of the federal poverty level [68].

Variations in the measurement of FI and social vulnerability and the characteristics of each studied sample made comparisons challenging. The varying prevalence of FI reported between studies is due to differences in measures, population characteristics, and country and local context. This bias cannot be accounted for in this review and is compounded by the difficulty in accessing hard-to-reach population subgroups, high research costs, and respondent burden associated with these types of surveys.

Factors identified in this review were categorized against an SEM to systematically depict the complexity of problems at proximal and distal levels to inform potential solutions [55]. SEM asserts that children function within a system outside of their individual, parental, and household characteristics, which are influenced by socio-environmental vulnerability [47]. Identifying factors across the system could support policy deliberations to identify appropriate interventions directed at each level.

Despite inconsistent measures of income or poverty and CFI, findings suggest that children from lower income (household factor) and poor households (societal factor) are more likely to experience CFI, consistent with previous research [45,46,101]. An important finding of this current review is the association of social vulnerability factors with the severity of CFI. Although vulnerability to CFI and low-income and poverty overlap and are used in some developed countries to estimate FI prevalence [37,102], they are not identical. Other key social vulnerability factors identified in this review include individual child factors (e.g., child’s age and sex), parental factors (e.g., parental depression, family stress), household factors (e.g., household composition, number of young children in the household), community factors (e.g., social cohesion), and societal factors (e.g., ethnic minority, housing). Further research is needed to explore the influence of these factors on CFI independent of poverty and income and relevant policy options to address them.

There were some inconsistencies in the association between social vulnerability factors and CFI, such as the age of the child. Older children were more likely to report FI in some studies but less likely or showed no significant relationship in others. The higher likelihood of FI in families with younger children may be due to less participation of mothers with infants or very young children in the labor force [103]. These findings suggest that the association between the child’s age and FI may be a curvilinear correlation and warrants further investigation.

The association between mental health and FI in people residing in households with children is likely to be a reverse causality. FI can be a traumatic experience, exacerbating mental health conditions. The 3 prospective studies included in this review found that maternal depression at baseline determined FI in households with children at follow-up [65,86,93]), and household FI at baseline was also related to maternal depression at follow-up, suggesting a bi-directional relationship.

The concept of ethnic minority (societal factor) was defined in many ways, e.g., parental country of birth, current immigration status, language spoken at home other than the national language, and parental nativity. Findings related to migration status, ethnic minorities, and CFI need to be interpreted with caution, as not all migrants are from minority groups. For example, unlike in most studies, Ramsey et al. [17] (2011) study of Australian children found those with a parent born outside of the country were less likely to experience FI, perhaps due to the largest group of immigrants in Australia being born in the UK and less likely to experience financial hardship [104]. Further, Australia’s migration program is highly selective of migrants with a higher SES, the majority comprising skilled migrants, which may explain this effect [105,106].

The diverse range of social vulnerability factors identified that are associated with CFI challenges current responses and supports, highlighting a comprehensive systems approach. Overall, the findings of the current review support the addition of FI as 1 of the adverse childhood experiences, a childhood condition that is consistently related to various long-term negative consequences [107]. The current review findings suggest that exposure to social vulnerability factors over long periods may be associated with chronic FI. Social vulnerability itself can subject children to social discrimination and isolation, which, in turn, can aggravate disadvantage [108].

### Recommendations for future research

Several issues hindered the ability to statistically determine the effect of each social vulnerability factor on CFI. First, inconsistent measures of both social vulnerability factors and CFI made direct comparison impossible. Future studies should use consistent and comparable measures of CFI and measure the severity and persistency of the problem. The USDA’s 18-item HFSSM measures the extent and severity of FI at the household level, including children, and has been validated and translated for use in many countries and population subgroups [[98], [109]]. The United Nations’ 8- and 10-item Food Insecurity Experience Scale promoted as the global FI index is validated but does not measure the experience of children [110].

Second, there is a dearth of research on CFI and its association with social vulnerability in economically developed countries beyond the United States and Canada [37,111] (where 39 of the 49 studies were conducted. Regular and robust monitoring and surveillance are critical research gaps in Europe and Australasia, where most studies have examined disadvantaged subgroups. Routine FI and societal vulnerability monitoring and surveillance systems focusing on children are lacking [37,111]. Critics suggest this may be due to the abdication of responsibility of the government to the third sector, who have fewer resources to conduct research [37,111]). Each country’s social protection context differs, reinforcing the need for intracountry monitoring and surveillance as well as across-country comparison. The lack of high-quality research investigating social vulnerability and the extent, severity, and persistence/trajectories of FI in children in most countries other than North America is concerning and warrants attention.

Third, most studies are cross-sectional, and few studies have investigated the association between social vulnerabilities and severity of FI in children, and no study has examined the association between persistent FI in children and its impact at different developmental stages, which is an important research gap. Well-designed longitudinal studies are urgently needed in countries other than the United States and Canada.

Fourthly, few studies asked children directly about their FI experience [52,[56], [58],^61^]. Development of effective interventions requires information about children’s perspectives on their own experience and conceptualization of FI, their roles within the household, how they make sense of their environments, and the social vulnerability they experience [112]. Younger children might not be able to speculate on the correlates of FI impacting their households; however, older children and adolescents can [113]. Developing a tool to assess older children’s experiences of FI and social vulnerability would provide an important but currently missing context.

Lastly, the COVID-19 global pandemic had pervasive socio-economic consequences that may impact social vulnerabilities and FI in children. Research is needed to investigate the impact of the COVID-19 pandemic on social vulnerabilities and FI and its impact on children to help inform emergency preparedness [113,114].

### Implications for public health interventions and policies

The current review highlighted social vulnerabilities as both drivers and consequences of CFI and the potential inadequacy of policy responses such as food assistance [115,116]. Social vulnerability factors can be used as candidate variables for the geographically based predictors of food stress [117], such as the food stress index, which guided food relief in response to the 2020 catastrophic Australian bushfires and COVID-19 [118]. However, an index predicting the geographic location of childhood social vulnerability to FI would foster more effective and equitable place-based solutions.

Social vulnerability starts at birth, and its impact accumulates over the course of life. To prevent and address social vulnerability and FI in children, it is important to create the conditions to support households with children to take control of their own lives, e.g., action across the identified social factors and beyond (Malmo’s framework) [119]. A collaborative approach led by governments involving private and voluntary organizations is recommended. The adopted SEM of social vulnerability and CFI could be used by decision-makers to identify leverage points for policy action.

The COVID-19 pandemic highlighted social vulnerability and FI, particularly among vulnerable households with children [62,120,121], and exposed the fragility of the food system, including food assistance and the financial security on which families rely [41]. Consequences included under-employment, school closures impacting feeding programs, and reduced household income [122]. The pandemic highlighted the critical role food charity programs play in most developed countries [13,123] but showed they fail to address chronic FI [13,41,124]. As Berg and Gibson [41] (2022) argue, “Charitable food distribution continues to grow, but it has done little to solve the problem [of FI].” Denying children the right to access sufficient, safe, and nutritious food in economically advanced countries with surplus food is indefensible and indicates a lack of political will, as “inequality is a political choice, not an inevitability” [125].

Finally, policy considerations should prioritize addressing CFI as it creates a substantial economic and social burden and contributes to healthcare costs [21,22,126]. The United States spends ∼A$179 billion each year due to FI and hunger [30,127]. Children living in FI households have greater rates of hospitalization [21,22,126] and emergency department visits [126]. There is much to be gained from taking action to minimize social vulnerability associated with CFI to prevent direct and indirect healthcare and other costs [128].

### Strength and limitations

A strength of this review is that it used a comprehensive and rigorous systematic methodological approach to identifying the association between social vulnerability factors and CFI at individual, proximal, and distal levels. These compiled data from high-income countries with comparable socio-economic positions, including North America, Europe, and Australasia, showed that FI is associated with an array of social vulnerability factors, highlighting unequal resource distribution in wealthy countries. To our knowledge, the current review is the first to comprehensively compile the key social vulnerability factors associated with CFI in economically developed countries across all regions. This review was also undertaken at a time when FI appears to be increasing as a public health issue across the globe, reinforcing the need to understand the social vulnerability factors influencing children.

There are some limitations; articles written in English only were included omitting studies published in other languages. In addition, most studies are cross-sectional, and the 12 longitudinal studies included in this review were conducted in North America. Outcomes of cross-sectional studies should be interpreted with caution due to the correlational nature of the analyses and the difficulty in determining whether factors are predictors or consequences of FI (e.g., the bilateral relationship identified between maternal depression and CFI) [86,93]. Another limitation is that due to the numerous instruments used to classify FS status (e.g., household, adult, child, or more generic) in a variety of contexts, direct comparison is not possible, and prevalence should be interpreted with scrutiny. Furthermore, the current study attempted to employ broad concepts and terms to identify social vulnerability and CFI; however, studies may have been missed, given the complexity of the issues and implications across a range of disciplines.

In conclusion, this systematic review identified social vulnerability factors associated with CFI in economically developed countries. Findings confirm income and poverty as social vulnerability factors associated with CFI, along with other factors such as individual child and parental socio-demographic factors, housing, household composition, and ethnicity. The association between parents’ mental health and physical health and CFI is less clear, and longitudinal research is warranted, as is research on CFI in developed countries other than the United States and Canada.

CFI prevalence in high-income countries was ≤96.5% in some sub-population segments, and several individuals, proximal (parental and household), and distal (community and societal) factors contributed to it. It is time to strengthen policies to reduce social vulnerability and protect children from the impact of FI.

## Author contributions

The authors’ responsibilities were as follows – LMD, CMP: conceived the study; LMD, CR-G: screened studies, extracted data, and conducted bias assessments; CMP, DAK: arbitrated assessments; LMD: drafted and edited the manuscript; CMP, DAK, JMF, JT, CMP: critically reviewed and edited the manuscript; and all authors: read and approved the final manuscript.

## Funding

The lead author, LMD, is supported through an Australian Government Research Training Program and Forrest Research Foundation Scholarship and undertook this work as part of her Doctor of Philosophy.

## Conflict of interest

The authors report no conflicts of interest.
