# Supplementary material for: Social Vulnerability and Child Food Insecurity in Developed Countries: A Systematic Review
Source: Adv Nutr. 2025 Jan 10;16(2):100365. doi: 10.1016/j.advnut.2025.100365 (PMC11847255; doi:10.1016/j.advnut.2025.100365)
Supplement: Multimedia component 1 [file mmc1.pdf]

Social vulnerability and child food insecurity in developed countries: A systematic review

Liyuwork M Dana

Supplementary Table 1. Search strategy concept grid on social vulnerability and food insecurity in Children: Social vulnerability and food insecurity among households with children in developed countries: A systematic review.

**CONCEPT 1**<br>**Food insecurity content Key terms**	**CONCEPT 2**<br>**Children Key terms**	**CONCEPT 3**<br>**Studies areas of food insecurity key terms**	**CONCEPT 4**<br>**Food insecurity factors key terms**
**OVID**<br>food insecur* OR food secur* OR hunger OR food desert* OR food adequa* OR food access* OR food avail* OR food utili*<br><br>Possible<br>*(food ADJ2 access* OR food ADJ2* avail* OR food *ADJ2* utili*)<br><br>*(food W/2 access* OR food W/2* avail* OR food *W/2* utili*)	child* OR<br>offspring* OR<br>toddler* OR<br>infant* OR<br>baby OR<br>babies OR<br>adolescen* OR<br>youth OR<br>teen* OR<br>Household with child* OR<br>Family with child* OR<br>childhood OR<br>pediatric	(Developed or industrialised or First world) adj2 (countr* or nation* or econom*)	social vulnerabili* OR<br>vulnerab* OR<br>social determinant* OR<br>determinant* OR<br>social class* OR<br>social factor* OR<br>social mobility OR<br>socio-economic*

		Austral* or<br>Austria or<br>Belg* or<br>Canad* or<br>Crotia* or<br>Cyprus* or<br>Romania* or<br>Czech* or<br>Denmark or<br>Danes* or<br>Estonia* or<br>Finland or<br>*Finnish* or*<br>France or<br>French* or<br>Germany or<br>Greece or<br>hunger* or<br>Iceland* or<br>Ireland* or<br>Irish* or<br>Ital* or<br>Japan* or<br>Latvia* or<br>Lithuania* or<br>Luxembourg* or	Netherland* or<br>Dutch* or<br>Malta* or<br>New Zealand* or<br>Norway or<br>Norwegian* or<br>Poland* or<br>Polish*<br>Portug* or<br>Slovak* or<br>Slovenia* or<br>Spain or<br>Spanish or<br>Swed* or<br>Switzerland or<br>Swiss* or<br>United Kingdom or<br>UK or<br>England or<br>Scotland or<br>Wales or<br>Ireland or<br>united states or<br>North America or<br>Europ*	income OR<br>*poverty* OR<br>educat*OR employ* OR<br>Gender OR<br>welfare-depend* OR<br>social depriv* OR<br>material depriv* OR<br>household compos* OR<br>household size OR<br>single parent* OR<br>minority OR<br>ethini* OR<br>material depriv* OR<br>finicial benef* OR<br>immigrant* OR<br>migra* OR<br>non-citizen* OR<br>social supp*
**MESH Medline**<br>exp Food Supply/	Exp Infant/ expo Child/exp Adolescent	As above listed countries	As above listed countries	exp Sociodemographic factors/<br>exp economic Factors

Social vulnerability and child food insecurity in developed countries: A systematic review

Liyuwork M Dana

Supplementary Table 2. Risk of bias assessment of included studies using the QualSyst tool* (*n*=46)

Study ID	Research Objective	Study Design	Sample Selection	Sample Detailed	Outcome Definition	Sample Size	Analytic Methods	Variance Estim.	Confounders Cont.	Results detailed	Conclusions	Total
Arteaga et al., 2017 (87)	2	2	2	2	2	2	2	2	2	2	2	22
Bhargav et al., 2008	2	2	2	2	1	2	2	2	2	2	1	20
Brewer et al., 2019(89)	2	2	2	2	2	2	2	2	2	2	2	22
Brewer et al., 2020(90)	2	2	2	2	2	2	2	2	2	2	2	22
Carter et al., 2012(76)	2	2	2	2	2	2	2	2	2	2	2	22
Denney et al., 2017(82)	2	2	2	2	2	2	2	2	2	2	2	22
Denney et al., 2020(91)	2	2	2	2	2	2	2	2	2	2	2	22
DeRigne et al., 2014(80)	2	1	2	2	2	2	2	2	2	2	2	21
Dhokarh et al., 2011(77)	2	2	1	2	2	1	2	2	2	2	1	19
Findlay et al., 2013(31)	2	2	2	2	2	2	2	2	2	2	2	22
Garg et al., 2015(92)	2	2	2	2	2	2	2	2	2	2	2	22

Gichunge et al., 2015(32)	2	2	1	2	2	1	1	2	1	2	1	17
Godrich et al., 2017(93)	2	2	2	2	2	2	2	2	2	2	2	22
Huet et al., 2017(94)	2	2	2	2	2	2	2	2	0	2	2	20
Ip et al., 2015(63)	2	2	2	2	2	2	2	2	2	2	2	22
Jolly et al., 2021(85)	2	1	1	1	1	1	1	1	0	1	2	12
Jomaa et al., 2020(95)	2	2	2	2	2	2	2	2	2	2	2	22
Kalil et al., 2008(96)	2	2	2	2	2	2	2	2	2	2	2	22
Kansanga et al., 2022(33)	2	2	2	2	2	2	2	2	2	2	2	22
Kowalski et al., 2021(73)	2	2	2	2	2	2	2	2	2	2	2	22
Lee et al., 2021(72)	2	2	2	2	2	2	2	2	2	2	2	22
Lippert et al., 2020(97)	2	2	2	2	2	2	2	2	2	2	2	22
Martin-Fernandez et al., 2018(64)	2	2	2	2	2	2	2	2	2	2	2	22

McIntyre et al., 2000(78)	2	2	2	0	2	2	2	2	2	2	2	20
McIntyre et al., 2002(84)	2	2	2	2	2	1	2	2	2	2	2	21
Melchior et al., 2009(71)	2	2	2	1	2	2	2	2	2	2	2	21
Miller et al., 2014(70)	2	2	2	2	2	2	2	2	2	2	2	22
Miller et al., 2018(69)	2	2	2	2	2	2	2	2	2	2	2	22
Morrissey et al., 2016(98)	2	2	2	2	1	2	2	2	2	2	2	21
Nagao-Sato et al., 2021(65)	2	2	2	2	2	1	1	2	2	2	1	19
Paquin et al., 2021(81)	2	2	2	2	2	2	2	2	2	2	2	22
Parekh et al., 2021(66)	2	1	2	2	2	2	2	2	2	2	1	20
Potochnick et al., 2019(99)	2	2	2	2	2	2	2	2	2	2	2	22
Ramsey et al., 2011(16)	2	2	2	2	2	1	2	2	2	2	2	21
Reesor-Oyer et al., 2021(100)	2	2	2	2	2	2	2	2	2	2	2	22
Rubio et al., 2019(101)	2	2	2	2	2	2	2	2	2	2	2	22

Ruiz-Castell et al., 2015(67)	2	1	2	2	2	1	2	2	2	2	2	20
Schlichting et al., 2018	1	2	2	1	1	2	1	2	2	2	2	18
Sharkey et al., 2011(79)	2	2	2	2	2	2	2	2	2	2	1	21
Utter et al., 2017(61)	2	2	2	2	1	2	1	2	2	1	1	18
Ward et al., 2020(68)	2	2	2	2	1	2	2	2	2	2	2	21
Wehler et al., 2004(102)	2	2	1	2	2	2	2	2	2	2	1	20
Wetherill et al., 2021(86)	2	2	2	2	2	1	2	2	2	2	2	21
Willis et al., 2020(62)	2	2	2	2	2	2	2	2	2	2	1	21
Zace et al., 2020(103)	2	2	2	1	2	2	2	2	2	2	2	21
Zhang et al., 2013(104)	2	2	2	2	2	2	2	2	2	2	1	21

Note: *QualSyst tool in which 11 checklists against the fulfilment of criterion for each of the articles were used, rating 2 for yes, 1 for partial, and 0 for not, with a maximum of 22 (2 points for each of the 11 criterion). The scores of articles included in this review ranges from 17 to 22.
